# Effects of planting date, environments and their interaction on grain yield and quality traits of maize hybrids

**DOI:** 10.1016/j.heliyon.2023.e21660

**Published:** 2023-10-30

**Authors:** Tesfaye Walle Mekonnen, Angeline van Biljon, Gert Ceronio, Maryke Labuschagne

**Affiliations:** a Department of Plant Sciences, University of the Free State, Bloemfontein, South Africa; b Department of Soil and Crop and Climate Sciences, University of the Free State, Bloemfontein, South Africa

**Keywords:** Environment, Maize, Protein, Planting dates, Starch

## Abstract

Maize is one of the most important staple food crops for most low-income households in the Southern African region. Erratic and inconsistent rainfall distribution across maize-growing areas is a major threat to maize production. Late rains in recent years have forced farmers to plant later than the optimal planting dates, leading to poor maize quality being reported by industry, which raised the question of the influence of later planting dates on grain yield and quality traits of maize. Three yellow and three white maize hybrids were evaluated at three planting dates in three different production environments for three consecutive seasons using a randomized complete block design with three replications. The second and third planting dates caused a significant yield decrease of 23.37 % and 53.73 % from the first planting date across environments, respectively. Planting date three was associated with decreased grain yield, starch content, and increased protein but no significant change in fat and fiber content. Some hybrids yielded relatively well at all planting dates. In conclusion, the early planting date was the most suitable for maize grain yield and starch production in the maize-growing areas of the country. However, planting in January should be avoided at all costs, as it leads to very low yield and poor grain quality.

## 1 Introduction

Maize (*Zea mays* L) is one of the most important food and forage staple crops, which has cultural, economic, environmental, and nutritional impacts worldwide [1,2]. It is the most widely cultivated cereal crop in the world and serves as a primary food source for nearly a billion people, predominantly in the developing world [3]. It provides food, feed, and nutritional security in the world's poorest regions in sub-Saharan Africa (SSA) Asia, and Latin America. At the same time, it is a major source of feed and industrial products in high-income countries [4].

In SSA, including South Africa, maize is a staple food crop and the most important human energy source with intakes ranging from 50 to >330 g/person/day and providing daily energy, protein, and micronutrients for low-income people in the region [2,5].

Climate change has been affecting food production globally through varying intensity and frequency of rainfall, the occurrence of extreme weather, and an increment in greenhouse gasses [6,7]. Environmental conditions have direct and significant impacts on maize growth and development throughout its growing season and, consequently, influence yield and seed quality [2,8]. The impact of climate change on maize yield, biomass, and nutritional quality traits has been significant [6]. Despite the many problems faced in maize production, the world still demands about 45 million tons of maize by 2030 for maintaining self-sufficiency [9].

Climate and environmental factors directly and significantly impact maize growth and development throughout its growing season, consequently influencing yield and seed quality [10]. Numerous climatic and environmental factors affect maize growth and development, including rainfall, radiation, and temperature [11,12]. The impact varies among cultivars because some maize cultivars rely more on temperature changes and less on photoperiod [11,12]. Temperature is critical in maize plant growth since cool temperatures slow growth and warmer temperatures accelerate growth and maturity [13,14].

Optimal crop planting date has the ability to reduce and eliminate the stress caused by insufficient water during crucial growth stages of crop development. This is important because the crop plating date has to be determined to minimise water stress throughout the entire growing period of the crop to reduce risk and increase grain yield significantly [10,15].

Therefore, determining planting dates for various maize cultivars is crucial for obtaining optimum crop yields since planting dates affect maize phenology [15]. Maize planted late is more exposed to adverse conditions like the early onset of frost, leading to reduced yields and poor seed quality. The responses of cultivars are different for different climate and environmental factors because of their genetic background. Hence, combining planting dates and cultivar selection can be a crucial production strategy for optimum crop growth and development, particularly under erratic environmental conditions.

Approximately 25 % of South Africa's total arable land (between 3.8 and 4.8 million ha) is planted with maize annually. In 2021/2022, the production of maize was approximately 15.3 million metric tons. The last five years' average annual commercial maize production was 14.94 million metric tons (https://www.statista.com/statistics/1134833/production-of-maize-in-south-africa/). White maize is used almost exclusively for human consumption and yellow for animal feed. The timing of maize plantings in South Africa depends on the onset of summer rainfall, which means early rains lead to early plantings, and late rains result in late plantings [16]. The optimum planting dates for maize in South Africa are from October to mid-December (www.nda.agric.za/docs/FactSheet/maize.htm). With late-onset rains, planting is postponed for weeks, with farmers sometimes planting as late as January. This study aimed to determine the effect of later planting on grain yield and quality in three production areas in South Africa compared to the optimal planting date.

## 2 Materials and methods

### 2.1 Description of experimental sites

**Fig. 1:**
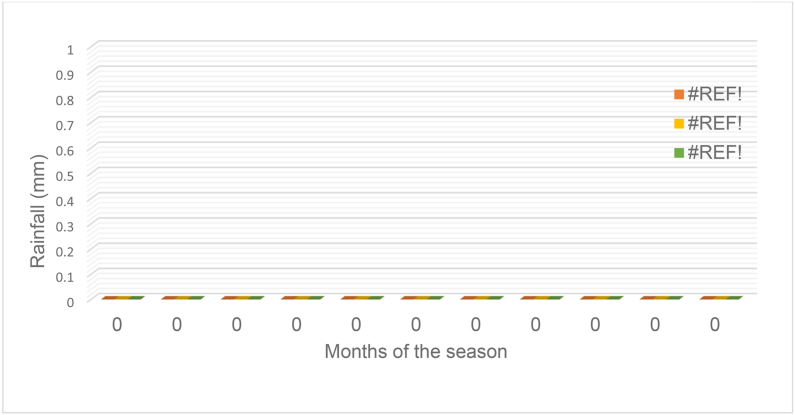
Rainfall distribution of Bloemfontein from 2019/2020–2021/2022 rainy season.

**Fig. 2:**
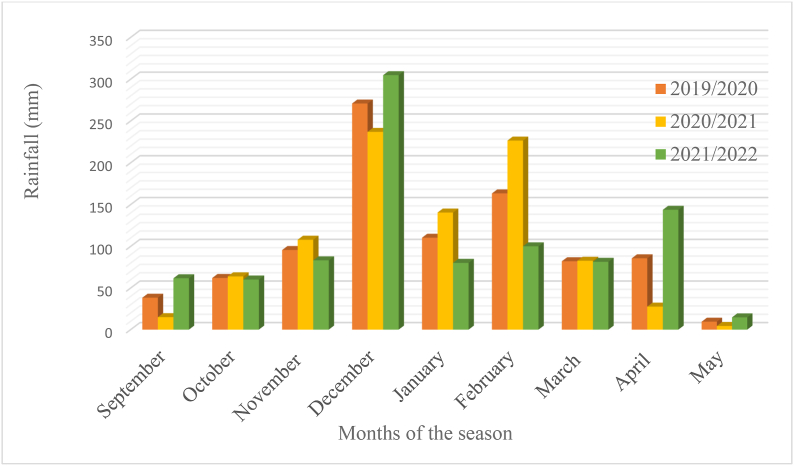
Rainfall distribution of Bethlehem from 2019/2020–2021/2022 rainy season.

**Fig. 3:**
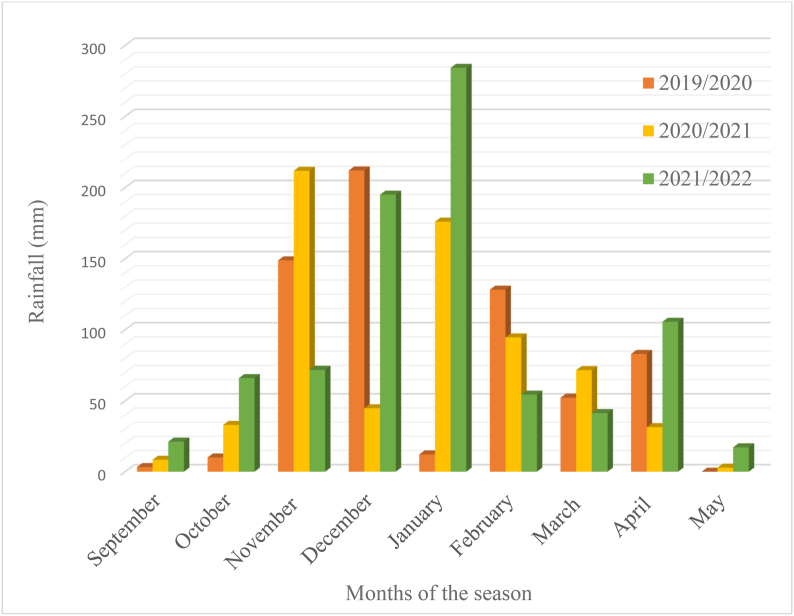
Rainfall distribution of Potchefstroom from 2019/2020–2021/2022 rainy season.

**Fig. 4:**
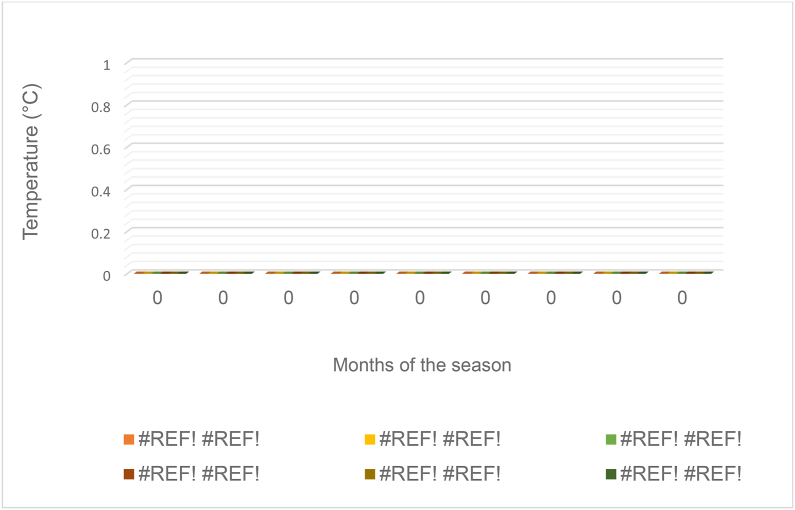
Monthly temperature distribution of Bloemfontein from 2019/2020–2021/2022 rainy seasons.

**Fig. 5:**
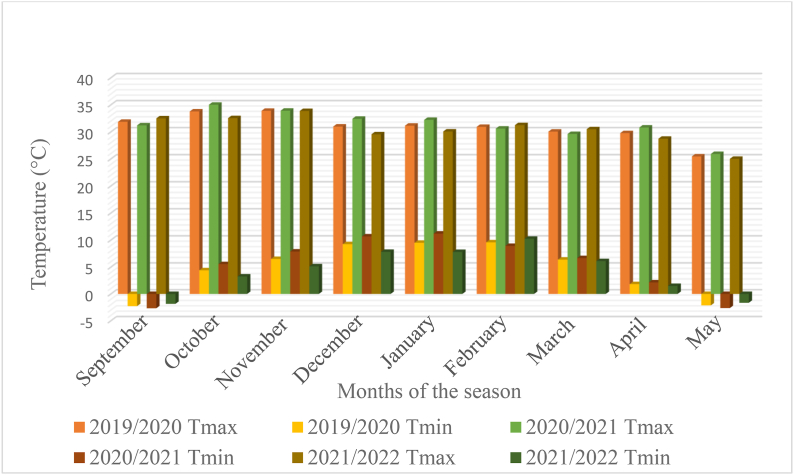
Monthly temperature distribution of Bethlehem from 2019/2020–2021/2022 rainy seasons.

**Fig. 6:**
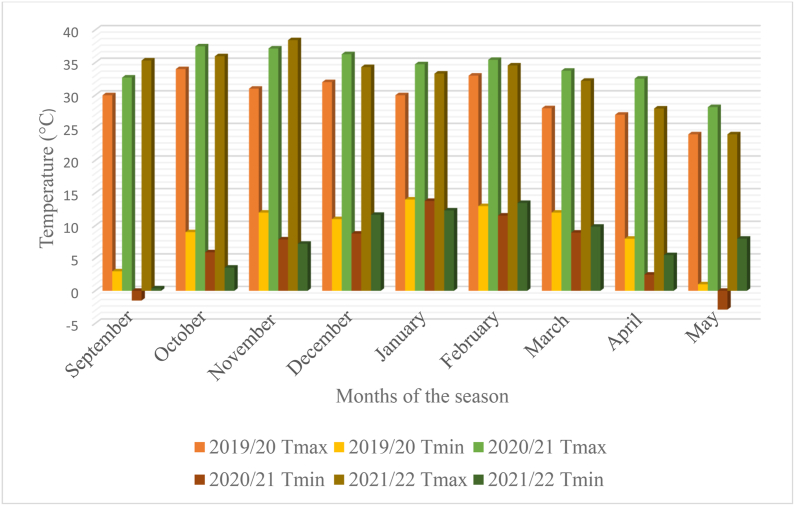
Monthly temperature distribution of Potchefstroom from 2019/2020–2021/2022 rainy seasons.

The trials were conducted during 2019/20, 2020/21 and 2021/2022 in the rainy season of South Africa at three environments namely Bloemfontein (sandy soil, altitude 1395 m.a.s.l., −29.02°N, 26.15°E), Bethlehem (sandy soil, altitude 1700 m.a.s.l., −28.16°N, 28.31°E) and Potchefstroom (sandy clay loam, altitude 1400 m.a.s.l., −26.72°N, 27.06°E). The test locations represent the major maize production regions of South Africa.The detailed decription of the study sites are presented in Table 1. The trails at each environment were established during the rainy (summer) season without irrigation supplement. The rainfall and temperature distributions are presented in Fig. 1, Fig. 2, Fig. 3, Fig. 4, Fig. 5, Fig. 6 during the experiment implementation time for the three consecutive seasons.

**Table 1:** Description of the study areas.

Location	Soil texture	Altitude (masl)	Latitude (N)	Longitude (E)	Annual Rainfall (mm)	T.max (°C)
Bloemfontein	Sandy	1395	−29.02°	26.15°	550	28.90
Bethlehem	Sandy	1700	−28.16°	28.31°	680	27.50
Potchefstroom	Sandy clay loam	1400	26.73°	27.06°	610	27.00

### 2.2 Plant materials

Six commercial maize hybrids namely DKC71-44B (yellow), DKC72-76BR (yellow), DKC74-26R (yellow), DKC75-65BR (white), DKC77-77BR (white) and DKC78-45BRGEN (white) were used in this study. These cultivars were selected from the available commercial hybrids in South Africa. Selection criteria included representation of cultivars grown throughout the summer rainfall region of South Africa (east to west), high yield probability and cultivar stability. The studied cultivars were intermediate maturity, and all cultivars were bred for dryland crop production areas of South Africa.

### 2.3 Experimental design and treatments

The experiments were laid out in a randomized complete block design (RCBD) with three replications. Each seed was planted at a depth of 5 cm. The distance between plants, rows and plot length was 60 cm, 91 cm and 10 m, respectively. Each seed was planted at a depth of 7 cm. Each plot consisted of four rows. At planting, 3:2:1 (25) 150 kg ha^−1^ was banded, and a top-dressing of 80 kg was applied three weeks after emergence. The range of the three planting dates for each year is given in Table 2. The first planting date (11–22 November) represented the recommended planting window for Bethlehem and Potchefstroom, and the second planting (10–20 December) the recommended for Bloemfontein, and the other two were roughly a month and two months later than the optimal planting window.

**Table 2:** Maize planting dates in each environment.

Environments	Planting dates	Actual planting dates		
		Season 1	Season 2	Season 3
		2019/2020	2020/2021	2021/2022
Potchefstroom	1	11 Nov 2019	20 Nov 2020	22 Nov 2021
	2	20 Dec 2019	18 Dec 2020	10 Dec 2021
	3	20 Jan 2020	20 Jan 2021	26 Jan 2022
Bethlehem	1	13 Nov 2019	17 Nov 2020	16 Nov 2021
	2	18 Dec 2019	15 Dec 2020	14 Dec 2021
	3	16 Jan 2020	19 Jan 2021	11 Jan 2022
Bloemfontein	1	19 Nov 2019	16 Nov 2020	15 Nov 2021
	2	17 Dec 2019	14 Dec 2020	13 Dec 2021
	3	15 Jan 2020	11 Jan 2021	10 Jan 2022

### 2.4 Data collection

#### 2.4.1 1 Grain yield

Grain yield data were obtained from the central two rows. The grain yield (kg ha^−1^) for every cultivar from the data of fresh weight per plot (adjusted to 12.5 % moisture) was calculated using the following formula [17]:

$$\text{Grain}\;\text{yield}\;\left(\text{kg}\;{\text{ha}}^{-1}\right)=\frac{Fresh\;ear\;weight\;\left(kg\;plot-1\right)\times \left(100-MC\right)\times 0.8\times 10,100}{\left(100-12.5\right)\times area\;harvested/plot}$$

where MC = moisture content in grams at harvest (%), 0.8 = Shelling coefficient, 10,000 m^2^ = 1 ha, 12.5 % = moisture content required in maize grain at storage.

#### 2.4.2 Protein, starch, crude fat, and crude fiber analyses

A total of 500 g of seeds for each sample was used for protein, starch, fat, and fiber content determination with Near-Infrared transmission spectroscopy (NIRS) using a Perten Grain Analyzer (Model DA 7250, Perten, Instruments AB, Sweden) in the 900–1700 nm wavelength range, with three subsamples for each sample. Results from wet chemistry analysis of 50 samples were used for confirmation of all values determined by the NIR. The correlation between the wet chemistry and the NIR values was more than 90 %, making the NIR values reliable (data not shown). The percentage of oil, protein, fat, and fiber contents were expressed on a dry matter percentage weight basis (% wt).

### 2.5 Statistical analyses

All grain yield and quality traits data were subjected to analysis of variance (ANOVA) using the randomized complete block design procedures described by Gomez and Gomez [18] with statistical analysis software [16]. The data of all test environments were subjected to the Shapiro-Wilk test for ANOVA residuals, which confirmed that data were normally distributed. The Hartley [19] test was used to test the heterogeneity of data from individual environments within the season and planting dates. According to the heterogeneity test, the data was homogenous, and the results confirmed that for combined analyses. The grain yield, starch, protein, fat and fiber data for three environments with three planting dates were analysed using general linear mixed model procedures with PROC MIXED in SAS, V9.4 software [20]. Hybrids were considered as a fixed factor, planting dates, environments, replication, and their interactions as random factors [21]. The means were separated by the least significance difference (LSD) procedure at 5 % significance levels. Although both yellow and white maize hybrids were included, preliminary analysis showed no effects from kernel color, hence, kernel color was not considered as a factor.

Principal component analysis (PCA) was performed after standardizing the variables using Minitab statistical software version 19 [22], a correlation matrix used for determining principal components, proportions of eigenvalues, and the scores of the principal components (PC).

## 3 Results

### 3.1 Analysis of variance

The combined ANOVA showed that grain yield was significantly (p < 0.01) affected by the main effects of season (S), hybrid (H), environment (E), planting dates (PD), as well as the interaction effects of E × PD, S × PD, and S × E × PD (Table 2). However, grain yield was not affected by the interaction of S × PD × H, S × PD × H and S × E × PD × H (Table 2). From grain quality traits, protein, starch, and fiber content were significantly affected by the main effects of season, environment, PD, H, E × PD, S × PD, and S × E × PD, but the protein, starch and fiber content were not affected by the interaction of S × PD × H, S × PD × H and S × E × PD × H (Table 3).

**Table 3:** Mean squares from analysis of variance for yield and quality traits for six hybrids over locations, seasons and planting dates.

Source of Variation	DF	Yield	Protein	Starch	Fat	Fiber
Season (S)	2	85756297**	4.14**	105.21**	2.50**	0.66**
Environment (E)	2	93887725**	27.26**	323.62**	0.46	0.20**
Replication	2	2544055	0.79	3.53	0.31	0.01
Planting dates (PD)	2	545919402**	65.87**	543.64**	0.20	1.94**
Hybrids (H)	5	5219963*	3.41**	33.68**	1.24**	0.16**
PD*H	10	3269769*	0.19	3.12	1.03**	0.02
E*PD	4	57225642**	37.74**	200.86**	1.11**	0.14**
S*PD	4	54701087**	32.45**	189.40**	0.63	0.49**
S*H	10	608980	0.60	4.22	0.83**	0.02
E*H	10	1230521	0.57	4.91*	0.47	0.02
E*PD*H	20	1498616	1.15**	6.57**	0.68**	0.02*
S *E*PD	7	33168771**	31.75**	141.89**	0.44	0.04**
S *E*H	20	1454352	0.57	3.42	0.39	0.02
S *PD*H	20	512515	0.61	3.87	0.30	0.01
S *E*PD*H	35	1624500	0.43	2.67	0.38	0.01
Error	202	1714374	0.47	2.46	0.29	0.02
R^2^		0.84	0.88	0.99	0.54	0.99
CV		26.17	8.29	2.41	12.46	4.12

*P ≤ 0.05, **P ≤ 0.01, CV = coefficient of variation, DF = degree of freedom, R^2^ = coefficient of determination.

### 3.2 Effect of planting dates, environments and their interaction on grain yield and quality traits

Planting dates and environments significantly affected grain yield, starch, protein, fat and fiber content in the three seasons (Table 4). At all three environments PD3 yielded significantly less than PD1 and PD2. Overall PD2 yield was lower than that of PD1 by 23.37 %, but PD3 yield was 53.73 % lower than PD1. For an unknown reason, starch content was significantly lower at Potchefstroom, and protein and fat content was significantly higher at PD2 than at PD1 and PD3. For the other two environments, starch was reduced considerably at PD3, and protein, fiber and fat increased at PD3. Across the environments, starch decreased by 0.75 % and 1.22 % at PD2 and PD3, and protein content increased by 1.89 % and 2.83 % for PD2 and PD3, compared to PD1, respectively. Compared to the other studied quality traits fat content was not affected by planting date overall.

**Table 4:** Grain yield, and starch, protein, fat and fiber content at three locations and three planting dates.

Environments	PD	Yield (kg ha^−1^)	Starch %	Protein %	Fat %	Fiber %
Potchefstroom	1	7051.00	64.22	7.68	4.30	2.60
	2	5337.07	60.31	9.18	4.49	2.59
	3	5799.54	61.69	8.62	4.20	2.75
Mean (kg ha^−1^)		6062.54	62.07	8.50	4.33	2.65
LSD		2416.89	3.66	1.50	0.60	0.25
Bloemfontein	1	6366.49	64.93	7.87	4.51	2.53
	2	5170.14	65.15	7.55	4.21	2.48
	3	2516.83	63.34	8.06	4.21	2.70
Mean (kg ha^−1^)		4379.79	64.47	7.83	4.31	2.57
LSD		481.08	1.77	0.78	0.64	0.16
Bethlehem	1	6910.52	63.96	7.88	4.23	2.49
	2	5069.56	63.78	7.74	4.34	2.59
	3	1089.40	57.58	10.26	4.21	2.65
Overall mean (kg ha^−1^)		4406.58	61.77	8.63	4.26	2.63
LSD		402.89	3.15	1.27	0.60	0.15
Overall mean PD1		6776.00	64.37	7.81	4.35	2.54
Overall mean PD2		5192.26	63.08	8.16	4.35	2.55
Overall mean PD3		3135.26	60.87	8.98	4.21	2.70
Overall mean (kg ha^−1^)		5034.51	62.77	8.32	4.30	2.60
CV%		21.85	4.56	14.22	14.26	7.18

CV = coefficient of variation, LSD = least significant difference, PD = planting date.

### 3.3 Cultivar performance across environments and planting dates

The mean grain yield ranged from 4265.04 kg ha^−1^ (DKC71-44B (H1) to 8462.16 kg ha^−1^ (DKC75-65BR (H6) at Potchefstroom across planting dates (Table 4). Hybrid performance was significantly affected by planting dates. The highest mean grain yield was obtained at PD1 at 8462.16 kg ha^−1^ (DKC75-65BR, H6). The lowest mean grain yield was obtained at PD3 (5337.07 kg ha^−1^) in this environment.

At Bloemfontein, the grain yield ranged from 1889.03 kg ha^−1^ to 7125.09 kg ha^−1^ with a mean of 4684.49 kg ha^−1^ at the first planting date (Table 5). In this environment, the lowest grain yield was 5456.58 kg ha^−1^, (DKC74-26R, H5), 4652.62 kg ha^−1^ (DKC72-76BR, H3), and 1889.03 kg ha ^1^ (DKC77-77BR, H2) had the highest grain yield at PD1, PD2 and PD3, respectively. DKC77-77BR (H2) (1498.48 kg ha^−1^) produced the lowest grain yield on the last (PD3) planting date but it produced less grain yield than the rest of the planting dates this indicates that the performance of hybrids is significantly affected by plant dates in this environment (Table 5).

**Table 5:** Average grain yield and starch, protein, fat and fiber content of six hybrids at three locations across two seasons.

Potchefstroom							Bloemfontein					Bethlehem				
PD	Entry	Yield	Starch	Protein	Fat	Fiber	Yield	Starch	Protein	Fat	Fiber	Yield	Starch	Protein	Fat	Fiber
	H1	5969.28	62.73	8.29	4.57	2.66	6453.69	63.86	7.90	5.11	2.64	6214.99	62.23	8.36	4.52	2.66
	H2	6943.14	64.75	7.44	4.04	2.52	7125.09	65.92	7.68	4.04	2.48	7459.28	64.61	7.70	4.07	2.45
1	H3	6774.68	63.88	7.68	4.55	2.63	6627.22	64.38	7.96	4.88	2.53	6753.81	63.50	8.01	4.53	2.50
	H4	6822.68	65.08	7.50	4.10	2.53	6234.12	65.55	7.76	4.53	2.46	6737.88	64.60	7.67	4.21	2.46
	H5	7328.04	63.84	7.99	4.29	2.66	5456.58	64.44	7.98	4.55	2.51	6693.89	63.72	7.98	4.33	2.45
	H6	8462.16	65.06	7.19	4.25	2.63	6302.27	65.42	7.96	3.96	2.56	7603.26	65.08	7.54	3.71	2.51
	Mean	7050.00	64.22	7.68	4.30	2.60	6366.49	64.93	7.87	4.51	2.53	6910.52	63.96	7.88	4.23	2.51
	LSD	2793.18	1.62	0.57	0.54	0.26	1138.26	1.91	0.91	0.75	0.11	1944.14	1.35	0.50	0.56	0.09
	H1	6046.79	61.44	8.47	4.06	2.61	5070.85	64.38	7.79	4.44	2.57	5200.90	62.99	8.02	4.37	2.68
	H2	5958.10	61.54	8.60	4.50	2.55	5002.86	65.86	7.35	4.05	2.43	5173.70	65.35	7.40	3.99	2.57
2	H3	5329.44	61.02	8.82	4.61	2.60	4652.62	65.33	7.38	4.31	2.54	4485.10	61.96	8.16	4.40	2.71
	H4	5381.05	62.18	8.63	4.98	2.62	5445.24	65.68	7.50	3.95	2.38	5285.24	64.81	7.26	4.47	2.57
	H5	5994.99	61.61	8.79	4.72	2.58	5240.60	64.59	7.71	4.40	2.43	4166.58	63.06	8.21	4.39	2.63
	H6	6086.86	62.37	8.43	4.07	2.56	5608.68	65.06	7.57	4.11	2.51	6005.52	64.52	7.40	4.44	2.69
	Mean	5799.54	61.69	8.62	4.49	2.59	5170.14	65.15	7.55	4.21	2.48	5052.84	63.78	7.74	4.34	2.64
	LSD	2474.95	3.32	1.26	0.72	0.27	749.04	1.54	0.85	0.58	0.13	1991.26	2.49	1.03	0.60	0.15
	H1	4265.04	59.15	9.49	4.06	2.71	2904.76	62.60	8.47	4.27	2.81	1438.69	57.32	10.34	4.22	2.73
	H2	5554.48	61.29	8.84	4.38	2.74	1889.03	63.79	7.88	4.18	2.66	828.03	59.62	9.53	4.27	2.65
3	H3	5027.13	60.48	9.07	4.45	2.79	2599.94	64.36	7.70	4.19	2.68	981.73	56.96	10.38	4.11	2.79
	H4	5960.27	59.53	9.60	4.15	2.68	2291.00	63.80	7.76	4.14	2.63	832.42	56.92	10.51	4.58	2.80
	H5	4972.23	60.42	9.07	4.09	2.78	2762.85	63.10	8.33	4.40	2.71	1492.87	57.38	10.53	3.81	2.74
	H6	6243.30	60.98	9.02	4.08	2.79	2653.38	62.37	8.19	4.07	2.72	962.68	57.27	10.30	4.24	2.81
	Mean	5337.07	60.31	9.18	4.20	2.75	2516.83	63.34	8.06	4.21	2.70	1089.40	57.58	10.26	4.21	2.75
	LSD	2047.70	6.29	2.77	0.39	0.25	1324.99	1.57	0.58	0.46	0.21	633.57	5.12	2.16	0.52	0.19
Overall mean		6062.20	62.07	8.49	4.33	2.65	4684.49	64.47	7.83	4.31	2.57	4350.92	61.77	8.63	4.26	2.63
CV		40.23	6.03	18.05	12.70	9.82	22.86	2.60	9.97	13.84	5.84	35.00	4.83	14.26	13.15	5.44

CV = coefficient of variation, H = cultivar, PD = planting date, LSD = least significant difference, H1 = DKC71-44B, H2 = DKC77-77BR, H3 = DKC72-76BR, H4 = DKC78-45BRGEN, H5 = DKC74-26R, H6 = DKC75-65BR

At Bethlehem, average grain yield varied from 828.03 kg ha^−1^ to 7603.26 kg ha^−1^. Based on the overall cultivar yield potential, PD1 was the best planting date in this environment. The lowest yield was obtained at PD3 (Table 4). DKC75-65BR (H6) (7603.26 kg ha^−1^) was the highest yielding cultivar at PD1 followed by DKC77-77BR (H2) (7459.28 kg ha^−1^), whereas the lowest yielding cultivar across planting dates was DKC77-77BR (H2) (828.03 kg ha^−1^).

Overall, average protein content varied from 7.19 % (Potchefstroom) to 8.36 % (Bethlehem) for PD1, 7.26 % (Bethlehem) to 9.60 % (Potchefstroom) for PD2 and 7.70 % (Bloemfontein) to 10.53 % (Bethlehem) for PD3. DKC78-45BRGEN had high protein content in Potchefstroom (9.60 %) and DKC71-44B (H1) 8.47 % in Bloemfontein and DKC74-26R (H5) 10.53 % in Bethlehem (10.53 %). Across the tested environments, DKC74-26R (H5) had the lowest protein content (7.19 %) while DKC75-65BR (H6) had the highest protein content (10.53 %).

### 3.4 Correlation of grain yield and quality traits in maize hybrids

At Potchefstroom, grain yield was positively correlated with starch content, and negatively correlated with protein, fat and fiber content. Significant and negative correlation between starch and protein content was seen, as expected (Table 6). At Bloemfontein, the grain yield was positively correlated with protein and fiber content. Fat and fiber content significantly and negatively correlated with starch content. Protein content was positively correlated with fat content (Table 6). At Bethlehem, grain yield was negatively correlated with protein, fat, and fiber content. Hence, starch content also negatively correlated with protein, fat, and fiber content.

**Table 6:** Simple correlation coefficients among five studied traits in maize hybrids.

Potchefstroom (A)					
Traits	Yield	Starch	Protein	Fat	Fiber
Starch	0.91*				
Protein	−0.83*	−0.90*			
Fat	−0.51	−0.25	0.36		
Fiber	−0.11	−0.43	0.30	0.03	
Bloemfontein (B)					
	Yield	Starch	Protein	Fat	Fiber
Starch	−0.23				
Protein	0.18	−0.97**			
Fat	−0.26	0.65	0.51*		
Fiber	0.55	−0.82*	0.69	0.57	
Bethlehem (C)					
Traits	Yield	Starch	Protein	Fat	Fiber
Starch	0.64				
Protein	−0.70	−0.96**			
Fat	−0.58	−0.60	0.46		
Fiber	0.04	−0.73	0.57	0.42	
Across environments (D)					
Traits	Yield	Starch	Protein	Fat	Fiber
Starch	0.60				
Protein	−0.62	−0.96**			
Fat	−0.91*	−0.74	0.71		
Fiber	−0.08	−0.81	0.65	0.31	

Grain yield positively correlated with starch content, and negatively correlated with protein, fat, and fiber content. Starch content was negatively correlated with protein, fat, and fiber content across the environments and seasons (Table 6).

### 3.5 Principal component analysis

The principal component analysis reflects the importance of the largest contributor to the total variation at each axis. In the present investigation, only the first two principal components having eigenvalues greater than one and cumulatively explaining 95.30 % of the total variation in the dataset were considered (Table 7). The first principal component (PC1) alone explained 72.40 % of the total variation. Protein, fat, and fiber content contributed positively to this PC1, while grain yield and starch contributed negatively. Grain yield and fiber content contributed positively to PC2, which had an eigenvalue of 1.15 and contributed of 22.90 % of the total variation in PC2 between the hybrids. However, fat content contributed negatively to PC2.

**Table 7:** Eigenvalue, the proportion of variance, cumulative variance and traits that contributed to the first two principal components for five traits of maize hybrids.

Traits	PC1	PC2
Yield	−0.41	0.58
Starch	−0.51	−0.23
Protein	0.49	0.13
Fat	0.46	−0.39
Fiber	0.35	0.67
Eigenvalue	3.62	1.15
The proportion of variance (%)	72.40	22.90
Cumulative (%)	72.40	95.30

PC = principal component.

**Fig. 7:**
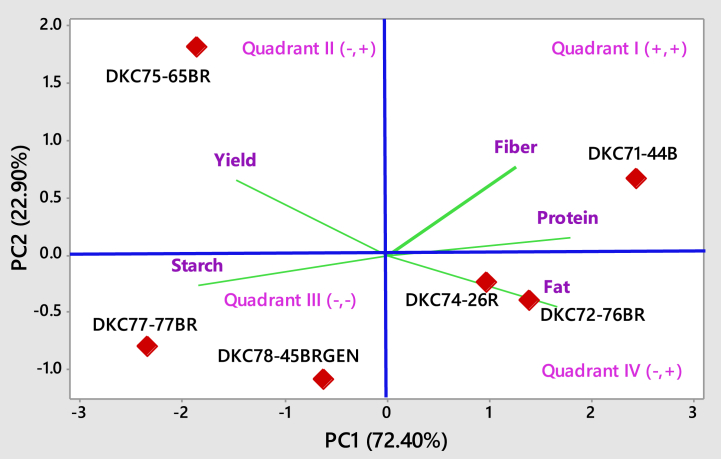
Loading plot showing association between five quantitative and qualitative traits of hybrids.

Hybrid DKC71-44B was in quadrant I, with high and positive values of protein and fiber content contribution comes from DKC71-44B for discrimination of the traits of the tested hybrids into four different quadrants. Those traits had a strong correlation and positively contributed to the discrimination of hybrids. In the third and fourth quadrant the hybrids were strongly related with starch, and fat content, respectively (Fig. 7). Hence, in quadrant two, DKC75-65BR correlated higher with grain yield. Fiber and protein overlapped in the two principal axes, having similar phenotypic and/or genotypic expressions.

## 4 Discussion

Understanding the effect of inconsistent rainfall distribution is the primary breeding objective for developing low moisture stress tolerant varieties to mitigate the threat of frequent drought and associated stress across the maize production areas and tackle the malnutrition problems. In this study, significant effects were detected for the main effects of season, environment, planting dates, hybrid, and interaction effects of E × PD, S × PD, S × H, S × E × PD, and S × PD × H for grain yield. This indicates that different planting dates, environments and seasons, and their interactions directly influenced hybrid grain yield performance. Grain yield is governed by several minor genes (alleles) and is, therefore, highly influenced by environmental effects. Golla et al. [23] reported similar findings on environments, planting dates, and their interaction effects on the grain yield of maize hybrids. The significant hybrid effect for protein, starch, fat, and fiber at different planting dates over seasons suggested that there was variation between hybrids over the planting dates for these characteristics and that this should be taken into account when developing nutritionally rich maize for the dryland area of southern Africa. Different planting dates were also previously reported to affect grain yield and yield-related traits [24,25] significantly.In addition, the grain yield potential of maize hybrids and the yield quality are correlated and strongly influenced by environmental factors such as temperature, total rainfall, and water storage in the soil [[10], [11], [12]].

Scheduling the planting dates for each production environment is an important decision for addressing the low moisture stress problem in maize production areas, which comes from an uneven rainfall distribution in SSA countries. In this study, the performance of the hybrids for grain yield was affected by the environment, season, planting dates and their interactions. PD2 yield was lower than that of PD1 by 23.37 %, but PD3 yield was 53.73 % lower than PD1. Starch was decreased by 0.75 % and 1.22 % at PD2 and PD3, and protein content was increased by 1.89 % and 2.83 % by PD2 and PD3, against PD1, respectively. Fat content was not significantly affected by planting date, while there was a significant difference only between PD2 and PD3 for fiber content. These results indicated that PD3 had by far the most significant detrimental effect on all measured traits, especially on grain yield. Planting so late should be avoided because grain yield is highly susceptible to moisture stress, particularly during the reproductive stage of the maize crop, which is a critical stage that needs optimal moisture to exploit the genetic potential for producing high grain yield. Previously, similar results were reported by Rahimi Jahangirlou et al. [8] on maize and Gao et al. [26] on sorghum.

Furthermore, there was a large variation between the hybrids for grain yield and the other measured quality traits. The late or delayed planting date (PD3) leads to a decline in grain yield, which may be attributed to the short grain filling period and vegetative period and grain yield potential. The present results were in agreement with Ke and Ma [25] and Tsimba et al. [27] on maize, Meleha et al. [25] on wheat. Moreover, the overall grain yield ranged from 828.03 kg ha^−1^ (PD3) to 8462.16 kg ha^−1^ (PD1) over seasons and locations, indicating that the late planting date should be avoided. However, high protein content (10.53 %) was recorded at the third planting (Table 4). This means that when the maize crop experiences moisture stress, the protein content in the grain is significantly increased.

In general, across environments, it was clear that farmers should stick to the first two planting dates. The second planting date around the half of December had a significantly lower yield of about 23.37 % compared to the first, so farmers would have to calculate the risk of planting at this date. This indicates that the influences of temperature and rainfall or soil moisture were favorable for grain yield at the first planting date. The third planting date should not be considered. Potchefstroom was the highest potential environment for maize production, especially for planting dates one and two. This was due to high rainfall, good distribution, and optimum temperatures during the growing season. The evaluated maize hybrids exhibited high variations in grain yield. The third planting date also significantly reduced the starch content (although relatively much less than the yield) and increased the protein content, which will undoubtedly negatively affect the cooking quality of the maize. This could explain the reports of poor cooking quality from consumers following the two production seasons reported in this paper (personal communication with Grain South Africa). This also confirms that planting in January should not be considered by farmers. Overall, the planting date did not significantly affect the fat and fiber content. A previous study also reported that different planting dates and hybrids greatly influence the grain yield potential of maize [28].

Dealing with and understanding the magnitude of the correlation of grain yield with starch, protein, fat, and fiber content of maize hybrids in various environments under staggered planting dates over seasons is essential to give a clear picture of trait association (which is generally due to linkage), which could contribute to a more effective selection strategy. In this study, across environments over seasons, grain yield positively correlated with starch content and negatively correlated with protein, fat, and fiber content. Starch content was negatively associated with protein, fat, and fiber content. This implies that it is difficult to develop high grain yield with quality traits (fat, fiber, and protein content) in maize in the different planting windows and environments as there is a “dilution” effect as yield and starch content increase. Similar findings were reported by Jahangirlou et al. [8], Idikut et al. [29] (2009) and Al-Naggar et al. [30].

In the present study, the first two principal components had eigenvalues more than one and cumulatively explained 95.30 % of the total variation. As per the principle of Joseph et al. [31], the loading effect of the studied traits greater than ±0.3 was regarded as meaningful and significant, while according to Chatfield and Collins [32], principal components with eigenvalues of less than one were eliminated from the PCA because they were not substantial. The first principal component (PC1) alone explained 72.40 % of the total variation, with protein, fat, and fiber content contributing positively to this PC, while grain yield and starch contributed negatively. Grain yield and fiber content contributed positively to PC2, which had an eigenvalue of 1.15 and explained 22.90 % of the total variation in the dataset. However, the fat content made a negative contribution to PC2. Mubai et al. [33] reported similar results on maize hybrids. Similarly, Felix et al. [34] used PCA to detect the most dominant traits from the studied maize hybrids. The extent of differences and correlation among the studied traits, as explained by the loading plot (Fig. 1), exhibited the magnitude of the association among the studied traits. In the third and fourth quadrants, the hybrids were strongly related to starch, and fat content, respectively (Fig. 7). Those traits had a strong correlation and positively contributed to the discrimination of hybrids. Hence, in quadrant two, DKC75-65BR is more correlated with grain yield. Fiber and protein overlapped in the two principal axes, having similar phenotypic and/or genotypic expressions. In this biplot, the traits far from the origin (x, y) have higher loading and significantly influence the classification. In this study, all traits far from the origin have a higher loading effect of the traits. The hybrids which are far apart and distant from the origin (0:0) are inherently diverse, while the hybrids close and overlapping on the loading plot exhibited similar characteristics (Fig. 7).

## 5 Conclusions

In the present investigation, environments and planting dates showed highly significant effects on grain yield, starch, protein, fat, and fiber. Planting dates highly significantly affected all the studied traits, except for the fat content. Hybrid DKC75-65BR had the highest grain yield and starch content across environments. Potchefstroom was the highest potential maize production area, especially for planting dates one and two. This was due to high and regular rainfall distribution and optimum temperatures during the growing season, particularly at the vegetative and reproductive stages. Grain yield of hybrids from PD1 to PD3 was decreased while protein, fat, and fiber content increased from PD1 to PD3 across the environments. This indicates that the early planting date was the most suitable for maize grain yield and starch production in the maize-growing areas of the country. However, the second planting date is usually the recommended planting date. The third planting date (January) should not be considered at all, as it carries a huge yield penalty and causes inferior grain quality.

## Funding statement

This study was funded by Department of Science and Innovation (10.13039/100016962DSI) through the Technology Innovation Agency and the Maize Trust, South Africa.

## Data availability statement

Data will be made available on request.

## CRediT authorship contribution statement

**Tesfaye Walle Mekonnen:** Conceptualization, Data curation, Formal analysis, Investigation, Methodology, Software, Validation, Visualization, Writing – original draft, Writing – review & editing. **Angeline van Biljon:** Conceptualization, Formal analysis, Methodology, Writing – review & editing. **Gert Ceronio:** Conceptualization, Methodology, Project administration, Validation. **Maryke Labuschagne:** Conceptualization, Funding acquisition, Project administration, Supervision, Writing – review & editing.

## Declaration of competing interest

The authors declare that they have no conflict of interest.
